# The association of CHA_2_DS_2_-VASc score and carotid plaque in patients with non-valvular atrial fibrillation

**DOI:** 10.1371/journal.pone.0210945

**Published:** 2019-02-08

**Authors:** Luxiang Shang, Yang Zhao, Mengjiao Shao, Huaxin Sun, Min Feng, Yaodong Li, Xianhui Zhou, Baopeng Tang

**Affiliations:** Department of Pacing and Electrophysiology, The First Affiliated Hospital of Xinjiang Medical University, Urumqi, Xinjiang, China; University of Palermo, ITALY

## Abstract

**Objective:**

The aim of this study was to assess the association between CHA_2_DS_2_-VASc score and carotid plaques in patients with non-valvular atrial fibrillation (NVAF).

**Methods:**

We conducted a retrospective study including 3,435 NVAF patients who underwent carotid ultrasound examinations from January 2015 to December 2017.We collected the clinical data on the medical records system. Chi-square trend test was used to analyze trends between the prevalence of carotid plaques with an increasing CHA_2_DS_2_-VASc score. Univariate and multivariate logistic regression was also used to assess the association between carotid plaques and CHA_2_DS_2_-VASc scores. The area under the receiver operating characteristic (ROC) curve (AUC) was used to determine the optimal cutoff points of different CHA_2_DS_2_-VASc scores in NVAF patients.

**Results:**

NVAF patients with carotid plaques had higher CHA_2_DS_2_-VASc scores compared with patients who did not have carotid plaques (3.01±1.36 vs. 2.55±1.28, *P* < 0.05). In all participants, male participants and female participants, the prevalence of carotid plaques increased significantly as the CHA_2_DS_2_-VASc score increased (*P* for trend < 0.001). Multivariate logistic regression analysis demonstrated that for each 1-point increase in the CHA_2_DS_2_-VASc score, there was an associated 37% increase in the prevalence of carotid plaques. ROC curve analysis revealed that a CHA_2_DS_2_-VASc score ≥ 2 in male patients (sensitivity, 44.67%; specificity, 75.64%; AUC, 0.639) or ≥ 3 in female patients (sensitivity, 47.24%; specificity, 72.40%; AUC, 0.634) were associated with carotid plaques.

**Conclusion:**

The prevalence of carotid plaques in patients with NVAF was associated with the CHA_2_DS_2_-VASc score.

## Introduction

Atrial fibrillation (AF) is the most common sustained arrhythmia in clinical practice, and the risk of stroke in patients with AF is five-fold greater than that of non-AF[1, 2]. The CHA_2_DS_2_-VASc score (Congestive heart failure, Hypertension, Age ≥ 75 [doubled], Diabetes, Stroke [doubled], Vascular disease, Age 65 to 74, and Sex category [female]) is a simplified risk score, which contains seven clinical variables that predict stroke risk and guide anticoagulation therapy in patients with non-valvular AF (NVAF)[3].

Carotid plaque that are measured by ultrasound is a common, safe and inexpensive method for evaluating subclinical atherosclerosis owing to its shallow position and easy measurement. Recent studies have shown that addition of carotid plaque detection in the CHA_2_DS_2_-VASc score can better predict the occurrence of stroke in patients with NVAF[4, 5]. However, the 2016 ESC atrial fibrillation guideline does not recommend carotid ultrasound as a routine examination for patients with NVAF[6]. Moreover, routine carotid ultrasound examination for all NVAF patients would result in large medical expenses and resources consumption.

In previous studies, several components of the CHA_2_DS_2_-VASc score, such as advanced age, diabetes mellitus, and hypertension, have been shown to be risk factors for carotid plaque formation[7–10]. However, few studies investigated the correlation between the overall CHA_2_DS_2_-VASc score and carotid plaques in NVAF patients.

Therefore, we sought to design a cross-sectional study to explore the correlation between the overall CHA_2_DS_2_-VASc score and carotid plaques in NVAF patients. It is expected to use this well-known risk score to evaluate the presence of carotid plaque and to improve the positive rate of carotid ultrasound in NVAF patients.

## Methods

### Patient population

This study is a single-center, retrospective, cross-sectional study. A total of 3,435 patients with NVAF who underwent carotid ultrasound examinations due to different clinical indications in our hospital between January 2015 and December 2017 were respectively reviewed. Patients were excluded from our study if they suffered from valvular heart disease, had a history of carotid endarterectomy or carotid artery stent implantation, or suffered from significant carotid malformation. This study protocol was reviewed and approved by the Institutional Review Board of The First Affiliated Hospital of Xinjiang Medical University, and conformed to the principles and guidelines of the Declaration of Helsinki.

### Data collection

All the data were acquired from electronic medical records, including demographic characteristics, lifestyle, medical history, body examination and blood laboratory tests. Age and gender were attained from the patients’ Identification Card. Current smoking or drinking habits, education level and use of oral anticoagulants were determined by self-reporting. Height, weight, waist circumference (WC) and blood pressure were measured by standard and calibrated instruments. Body mass index (BMI) was calculated as weight (in kilograms) divided by height (in meters) squared. Fasting blood glucose (FBG), triglyceride (TG), total cholesterol (TC) and low-density lipoprotein-cholesterol (LDL-C) were obtained from blood laboratory test during hospitalization. The diagnosis of diseases was from the discharge diagnosis in the medical record system and diagnostic codes were in the format of the International Classification of Disease, 9th Revision, Clinical Modification.

### Assessment of carotid plaque

Carotid plaques were measured by certified and experienced sonographers using ultrasounds (Philips HD11XE ultrasound system, Philips Medical Systems, Bothell, WA). The frequency of the ultrasound probe was 7.5MHz. Both sides of the carotid arteries were extensively scanned. The patients were placed in a supine position, their heads were tilted towards the examination area, and the sonographer fully exposed one side of the neck. After checking one side, the head was turned to the opposite side and was observed in the same way. The common carotid arteries, carotid bifurcation, internal carotid artery and external carotid artery were all examined. Carotid plaques were defined as a focal structure that uplifted into the arterial lumen at least 0.5 mm or 50% of the surrounding intima-media thickness (IMT) value; or it was defined as an IMT greater than 1.5 mm. The results of carotid ultrasounds were reviewed by two independent operators and subsequently input into medical record system.

### Statistical analysis

The SPSS version 22.0 software (SPSS Inc., Chicago, IL) was used to analyze the data. Continuous data were presented as means ± standard deviation (SD) and were compared by *t*-test analysis. Categorical data were presented as proportions and were compared with Pearson Chi-square test. The Chi-square trend test was used to analyze the trends of the detection rate of carotid plaques with an increasing CHA_2_DS_2_-VASc score. Univariate and multivariate logistic regression was also used to assess the association between carotid plaques and the CHA_2_DS_2_-VASc scores. Education level, smoking, drinking, BMI, FBG, TG, TC, and LDL-C were adjusted in the multivariate logistic regression analysis. The area under the receiver operating characteristic curve (AUC) was used to determine the optimal cutoff points of different CHA_2_DS_2_-VASc scores in NVAF patients. All tests were two-tailed, and a *P* value <0.05 was considered statistically significant.

## Result

### Patient characteristics

Patients were divided into two groups based on detection of a carotid plaque or not: no carotid plaque group (n = 1,632) and with carotid plaque group (n = 1,803). The characteristics and clinical data of participants, in the presence or absence of carotid plaques, are presented in Table 1. Patients with carotid plaques were older and had higher levels of SBP, DBP, FBG, TC, LDL-C, as well as a higher CHA_2_DS_2_-VASc score; moreover, there was a higher prevalence in males, patients with a lower education level, those who were currently smoking and drinking, as well as patients using OACs (all *P* value < 0.05). There was no significant difference between the two groups in WC and TG.

**Table 1:** Demographic and clinical data according to the detection of carotid plaques.

Characteristics	No carotid plaque	With carotid plaque	*P* value
	(n = 1632)	(n = 1803)	
Age, year	59.14±9.50	64.91±9.32	<0.001
Male, n (%)	505 (30.9)	806(44.7)	<0.001
Education level, n (%)			<0.001
Primary school and below	817 (50.1)	1065(59.1)	
Middle school	551 (33.8)	495(27.5)	
High school or higher	264 (16.2)	243(13.5)	
Current smoking, n (%)	247 (15.1)	342(19.0)	0.003
Current drinking, n (%)	209 (12.8)	363(20.1)	<0.001
BMI, kg/m^2^	26.24±3.64	25.75±3.63	<0.001
WC, cm	88.16±11.90	88.84±11.44	0.135
SBP, mmHg	140.44±21.63	146.89±22.28	<0.001
DBP, mmHg	85.91±12.66	87.45±13.36	0.001
FBG, mmol/L	5.83±1.85	6.05±2.07	0.001
TG, mmol/L	1.70±1.17	1.74±1.09	0.290
TC, mmol/L	4.96±1.08	5.09±1.13	<0.001
LDL-C, mmol/L	2.90±0.94	2.97±0.93	0.026
OACs, n (%)	671 (41.1)	995 (55.2)	<0.001
CHA_2_DS_2_-VASc score	2.55±1.28	3.01±1.36	<0.001
Distribution of CHA_2_DS_2_-VASc score			<0.001
0 point	54 (3.3)	29 (1.6)	
1 point	273 (16.7)	209 (11.6)	
2 points	534 (32.7)	440 (24.4)	
3 points	413 (25.3)	484 (26.8)	
4 points	244 (15.0)	395 (21.9)	
5 points	88 (5.4)	180 (10.0)	
6 points or more	26 (1.6)	66 (3.7)	

BMI: body mass index, WC: waist circumference, SBP: systolic blood pressure, DBP: diastolic blood pressure, FBG: fasting blood glucose, TG: triglyceride, TC: total cholesterol, LDL-C: low density lipoprotein cholesterol; OACs: oral anticoagulants.

### The prevalence of carotid plaque stratified by the CHA_2_DS_2_-VASc score

**Fig 1:**
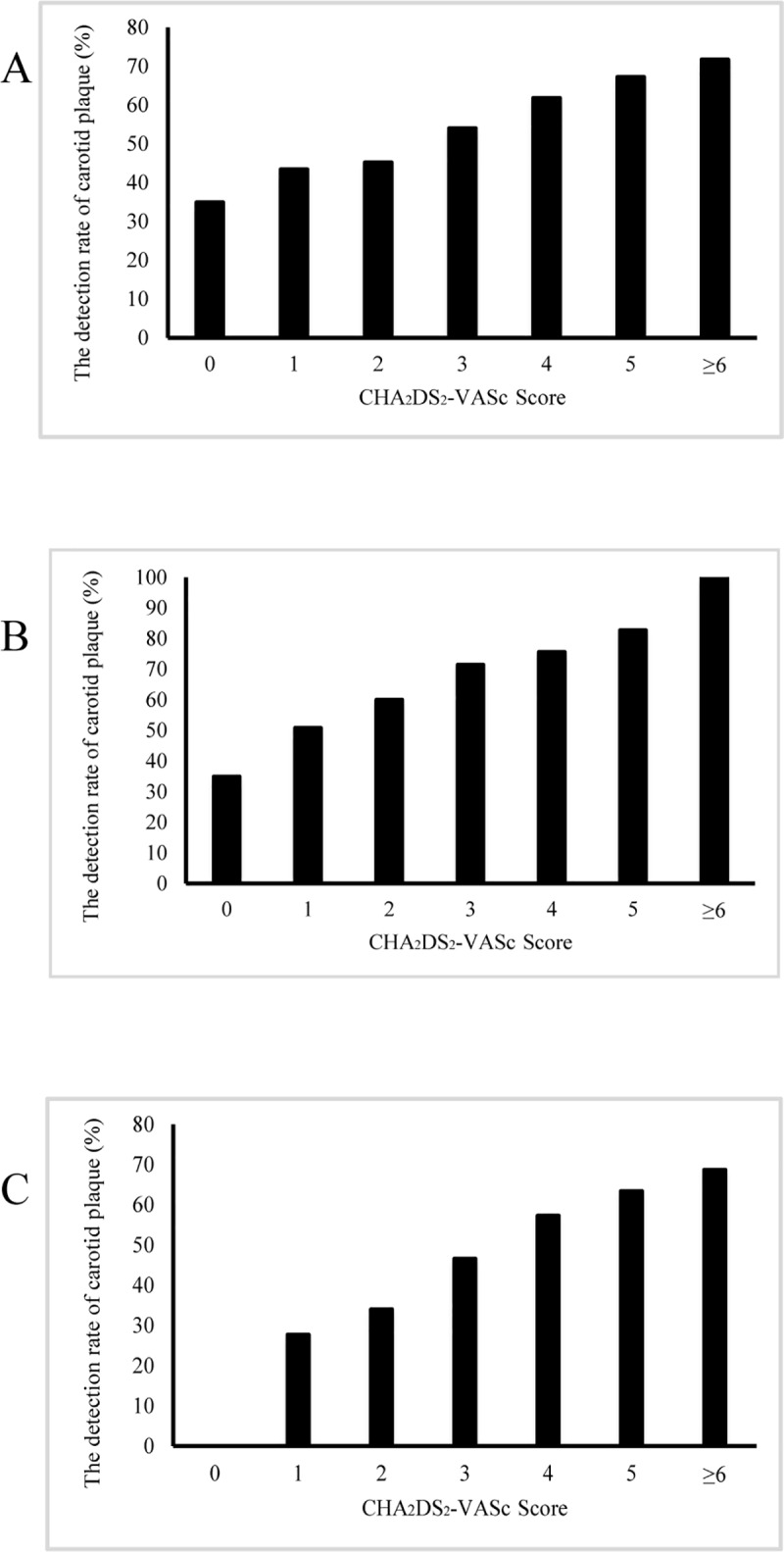
The prevalence of carotid plaque stratified by CHA_2_DS_2_-VASc score in NVAF patients. (A) In all patients (*P* for trend < 0.001). (B) In male patients (*P* for trend < 0.001). (C) In female patients (*P* for trend < 0.001).

As shown in Fig 1, the prevalence of carotid plaques were 34.9%, 43.4%, 45.2%, 54.0%, 61.8%, 67.2%, and 71.7% in patients with a CHA_2_DS_2_-VASc score of 0, 1, 2, 3, 4, 5 and ≥ 6, respectively. The prevalence of carotid plaque increased significantly with the increase of CHA_2_DS_2_-VASc scores in all participants, male participants and female participants (*P* for trend < 0.001).

### Evaluating the cutoff value of the CHA_2_DS_2_-VASc score to predict carotid plaque

**Fig 2:**
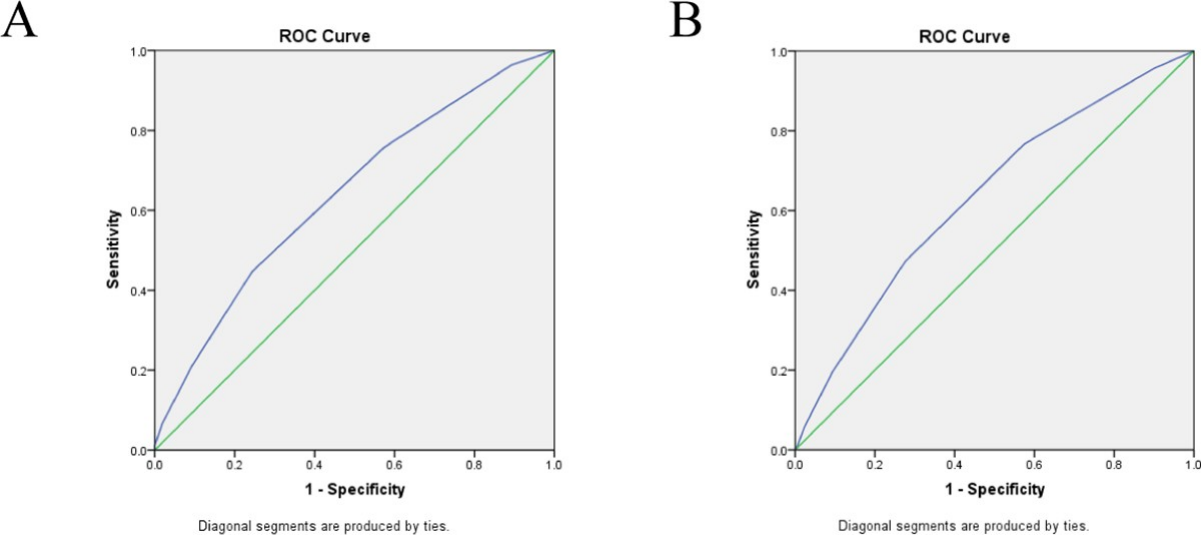
Receiver operating characteristic curves for CHA_2_DS_2_-VASc score for evaluation of carotid plaques in NVAF patients. (A) In male patients. (B) In female patients.

For male NVAF patients, a cutoff value of ≥ 2 for the CHA_2_DS_2_-VASc score to evaluate carotid plaques, which had a sensitivity of 44.67% and specificity of 75.64%, AUC: 0.639 with 95% CI (0.613–0.665). For female NVAF patients, a cutoff value of ≥ 3 for the CHA_2_DS_2_-VASc score when evaluating carotid plaque, which had a sensitivity of 47.24% and specificity of 72.40%, AUC: 0.634 with 95% CI (0.613–0.654) (Fig 2).

### Association of CHA_2_DS_2_-VASc score with carotid plaque

Table 2 showed that per 1-point increase in the CHA_2_DS_2_-VASc score, it was significantly associated with the prevalence of carotid plaque in all patients (OR: 1.304; 95% CI: 1.238–1.374); in male patients (OR: 1.537; 95% CI: 1.393–1.695) and in female patients (OR: 1.476; 95% CI: 1.372–1.588). Male NVAF patients who had a CHA_2_DS_2_-VASc score of ≥ 2 were 2.323-fold (95% CI: 1.829–2.950) more likely to develop carotid plaques compared with those who had a CHA_2_DS_2_-VASc score of < 2. While female NVAF patients with a CHA_2_DS_2_-VASc score ≥ 3 were 2.437-fold (95% CI: 2.019–2.943) more likely to develop carotid plaques than those with a CHA_2_DS_2_-VASc score < 3. In the multivariate logistic regression analysis, the CHA_2_DS_2_-VASc score was still a significant independent predictor of carotid plaques in both male and female NVAF patients.

**Table 2:** OR and 95% CI between CHA_2_DS_2_-VASc score and carotid plaque prevalence.

Group	Unadjusted model		Adjusted model*	
	OR (95%CI)	*P* value	OR (95%CI)	*P* value
Total study population				
Per 1-point increase	1.304 (1.238–1.374)	<0.001	1.370 (1.293–1.543)	<0.001
Male				
CHA_2_DS_2_-VASc score < 2	Reference		Reference	
CHA_2_DS_2_-VASc score ≥ 2	2.323 (1.829–2.950)	<0.001	2.429 (1.884–3.132)	<0.001
Per 1-point increase	1.537 (1.393–1.695)	<0.001	1.592 (1.431–1.770)	<0.001
Female				
CHA_2_DS_2_-VASc score < 3	Reference		Reference	
CHA_2_DS_2_-VASc score ≥ 3	2.437 (2.019–2.943)	<0.001	2.148 (1.766–2.613)	<0.001
Per 1-point increase	1.476 (1.372–1.588)	<0.001	1.410 (1.306–1.522)	<0.001

*Adjusted for education level, current smoking, current drinking, BMI, FBG, TG, TC, LDL-C.

OR: odds ratio, CI: confidence interval.

## Discussion

### Main finding

Our key findings were as follows: (1) CHA_2_DS_2_-VASc score was independently associated with carotid plaques in NVAF patients; (2) The optimal cutoff point of the CHA_2_DS_2_-VASc score for evaluating carotid plaques were 2 and 3 in male and female NVAF patients, respectively.

### High prevalence of carotid plaque in NVAF patients

AF and atherosclerosis may influence the development of each other[11]. As a marker of subclinical atherosclerosis, it is supposed that NVAF patients have relatively high prevalence of carotid plaque formation[12]. However, currently there is limited data pertaining to the prevalence of carotid plaques in NVAF patients.

In a recent subgroup analysis of Atherosclerosis Risk in Communities (ARIC) study, the prevalence of carotid plaques was 38.1% in 724 patients (average age 63.3 years, female 40.1%) who developed AF within 5 years and had no history of stroke[5]. In a cohort study, to investigate the association between carotid atherosclerosis and risk of ischemic stroke in AF patients on anticoagulant treatment, the prevalence of carotid atherosclerosis was 64.7% in 587 patients (average age 74.5 years, female 41.4%)[13]. In our study, 52.5% of NVAF patients were detected with carotid plaques. The prevalence of carotid atherosclerosis is increased with age[14]. Although the average age of participants in our study was similar to those in the ARIC study, but the prevalence of carotid plaques was much higher, suggesting that the prevalence of carotid plaques in Chinese NVAF patients might be higher.

### The importance of carotid ultrasound examination in NVAF patients

Stroke is the second leading cause of mortality and the third leading cause of long-term disability worldwide[15]. Furthermore, the prevention and management of ischemic stroke has become a heavy public health problem in both developed and developing countries[15, 16]. Identification of patients who are at high risk of stroke is important for providing the best treatment strategy and to improve the patients’ outcome.

NVAF patients with carotid plaques have a higher stroke risk. The result of the ARIC study indicated that the presence of carotid plaques was significantly associated with a 56% increase increased stroke risk[5]. The findings from of studies conducted by Becattini et al. showed that carotid atherosclerosis was associated with a significant increase in the risk for the composite of ischemic stroke or transient ischemic attack, or death after adjusting for the CHA_2_DS_2_-VASc score[13]. About one-third of AF patients suffered from a non-cardioembolic stroke, the risk of ischemic stroke due to carotid atherosclerosis is underestimated in NVAF patients[17–19].

Our study suggests that the prevalence of carotid plaques increase in NVAF patients as the CHA_2_DS_2_-VASc score increases. For NVAF patients with a high CHA_2_DS_2_-VASc score, routine carotid ultrasound examinations might be recommended. Furthermore, more individualized stroke prevention strategies in NVAF patients with carotid disease should be considered.

### Limitations

This study had several limitations. First, it was a single center, retrospective and observational study, and had inherent limitations of a retrospective design. Second, the study population was searched from electronic medical record system, and had selective bias. Third, the information of type of AF was lacking. Forth, some risk factors which had been verified to be related to carotid plaque were not included as the database, which might lead to some bias in multivariate logistic regression analysis. Further prospective investigations are needed to confirm this correlation.

## Conclusion

In conclusion, as the CHA_2_DS_2_-VASC score increased, the prevalence of carotid plaques increased in NVAF patients. Carotid ultrasound examinations may be more indicated in male NVAF patients with a CHA_2_DS_2_-VASc score ≥ 2 and female NVAF patients with a CHA_2_DS_2_-VASc score ≥ 3.

## Supporting information

S1 Data (XLS)

S1 Text STROBE checklist. (DOCX)

S2 Text Clinical studies checklist. (DOCX)
