# Supplementary material for: The association of CHA2DS2-VASc score and carotid plaque in patients with non-valvular atrial fibrillation
Source: PLoS One. 2019 Feb 8;14(2):e0210945. doi: 10.1371/journal.pone.0210945 (PMC6368281; doi:10.1371/journal.pone.0210945)
Supplement: S1 Text — (DOCX) [file pone.0210945.s002.docx]

STROBE Statement—checklist of items that should be included in reports of observational studies

|  | | | Item No. | Recommendation | Page  No. | | | Relevant text from manuscript |
| --- | --- | --- | --- | --- | --- | --- | --- | --- |
| **Title and abstract** | | | 1 | (*a*) Indicate the study’s design with a commonly used term in the title or the abstract | 1-2 | | | a retrospective study |
|  |  |  |  | (*b*) Provide in the abstract an informative and balanced summary of what was done and what was found | 1-2 | | | We conducted a retrospective study including 3,435 NVAF patients which performed carotid ultrasound examinations from January 2015 to December 2017.We collected the clinical data on the medical records system. |
| Introduction | | | | | | | |  |
| Background/rationale | | | 2 | Explain the scientific background and rationale for the investigation being reported | 4-5 | | | Atrial fibrillation (AF) is the most common sustained arrhythmia in clinical practice, and the risk of stroke in patients with AF is five-fold than that of non-AF. CHA2DS2-VASc score is a simplified risk score which contains seven clinical variables to predict stroke risk and guide anticoagulation therapy in patients with non-valvular AF (NVAF) |
| Objectives | | | 3 | State specific objectives, including any prespecified hypotheses | 4-5 | | | Therefore, we sought to design a cross-sectional study to explore the correlation between overall CHA_2_DS_2_-VASc score and carotid plaque in NVAF patients. It is expected to use this well-known risk score to predict the presence of carotid plaque and to improve the positive rate of carotid ultrasound in NVAF patients. |
| Methods | | | | | | | |  |
| Study design | | | 4 | Present key elements of study design early in the paper | 5 | | | This study is a single-center, retrospective, cross-sectional study. |
| Setting | | | 5 | Describe the setting, locations, and relevant dates, including periods of recruitment, exposure, follow-up, and data collection | 5-6 | | | A total of 3,435 patients with NVAF which performed carotid ultrasound examinations due to diﬀerent clinical indications at our hospital between January 2015 and December 2017 were respectively reviewed. |
| Participants | | | 6 | (*a*) *Cohort study*—Give the eligibility criteria, and the sources and methods of selection of participants. Describe methods of follow-up  *Case-control study*—Give the eligibility criteria, and the sources and methods of case ascertainment and control selection. Give the rationale for the choice of cases and controls  *Cross-sectional study*—Give the eligibility criteria, and the sources and methods of selection of participants | 5-6 | | | A total of 3,435 patients with NVAF which performed carotid ultrasound examinations due to diﬀerent clinical indications at our hospital between January 2015 and December 2017 were respectively reviewed. Patients with valvular heart disease, history of carotid endarterectomy or carotid artery stent implantation, significant carotid malformation were excluded. |
|  |  |  |  | (*b*) *Cohort study*—For matched studies, give matching criteria and number of exposed and unexposed  *Case-control study*—For matched studies, give matching criteria and the number of controls per case |  | | |  |
| Variables | | | 7 | Clearly define all outcomes, exposures, predictors, potential confounders, and effect modifiers. Give diagnostic criteria, if applicable | 5-6 | | | All the data were acquired on electronic medical records, including demographic characteristics, lifestyle, medical history, body examination and blood laboratory testing. |
| Data sources/ measurement | | | 8* | For each variable of interest, give sources of data and details of methods of assessment (measurement). Describe comparability of assessment methods if there is more than one group | 5-6 | | | Age and gender were recorded by Identification Card. Current Smoking, drinking habits and education level was determined by self-report. Height, weight, waist circumference (WC) and blood pressure were measured by standard and calibrated instruments, and body mass index (BMI) was calculated as weight (in kilograms) divided by height (in meters) squared. Fasting blood glucose (FBG), triglyceride (TG), total cholesterol (TC) and low density lipoprotein-cholesterol (LDL-C) were obtained from blood laboratory testing during hospitalization. The diagnosis of diseases were from the discharge diagnosis in the medical record system and diagnostic codes were in the format of the International Classification of Disease, 9th Revision, Clinical Modification. |
| Bias | | | 9 | Describe any efforts to address potential sources of bias | 14-15 | | | Second, the study population was searched from electronic medical record system, and had selective bias. Third, some risk factors which had been verified to be related to carotid plaque were not included as the database, which might lead to some bias in multivariate logistic regression analysis. |
| Study size | | | 10 | Explain how the study size was arrived at | 5 | | | A total of 3,435 patients with NVAF which performed carotid ultrasound examinations due to diﬀerent clinical indications at our hospital between January 2015 and December 2017 were respectively reviewed. |
| Quantitative variables | | 11 | | Explain how quantitative variables were handled in the analyses. If applicable, describe which groupings were chosen and why | 5-6 | | Current Smoking, drinking habits and education level was determined by self-report. The diagnosis of diseases were from the discharge diagnosis in the medical record system and diagnostic codes were in the format of the International Classification of Disease, 9th Revision, Clinical Modification. | |
| Statistical methods | | 12 | | (*a*) Describe all statistical methods, including those used to control for confounding | 7 | | SPSS version 22.0 software (SPSS Inc., Chicago, IL) was used to analysis the date. Continuous data were presented as means ± standard deviation (SD) and were compared by *t*-test analysis. Categorical data were presented as proportions and were compared with Pearson chi-square test. The chi-square trend test was used to analysis the trends of the detection rate of carotid plaque with increasing CHA_2_DS_2_-VASc score. Univariate and multivariate logistic regression was also used to assess the association between carotid plaque and CHA_2_DS_2_-VASc score. Education level, smoking, drinking, BMI, FBG, TG, TC, LDL-C were adjusted in the multivariate logistic regression analysis. The area under the receiver operating characteristic curve (AUC) was used to determine the optimal cutoff points of different CHA_2_DS_2_-VASc scores in NVAF patients. All tests were two-tailed, and a *P* value <0.05 was considered statistically significant. | |
|  |  |  |  | (*b*) Describe any methods used to examine subgroups and interactions |  | |  | |
|  |  |  |  | (*c*) Explain how missing data were addressed |  | |  | |
|  |  |  |  | (*d*) *Cohort study*—If applicable, explain how loss to follow-up was addressed  *Case-control study*—If applicable, explain how matching of cases and controls was addressed  *Cross-sectional study*—If applicable, describe analytical methods taking account of sampling strategy |  | |  | |
|  |  |  |  | (*e*) Describe any sensitivity analyses |  | |  | |
| Results | | | | | | | | |
| Participants | | 13* | | (a) Report numbers of individuals at each stage of study—eg numbers potentially eligible, examined for eligibility, confirmed eligible, included in the study, completing follow-up, and analysed | 5 | | A total of 3,435 patients with NVAF which performed carotid ultrasound examinations due to diﬀerent clinical indications at our hospital between January 2015 and December 2017 were respectively reviewed. | |
|  |  |  |  | (b) Give reasons for non-participation at each stage |  | |  | |
|  |  |  |  | (c) Consider use of a flow diagram |  | |  | |
| Descriptive data | | 14* | | (a) Give characteristics of study participants (eg demographic, clinical, social) and information on exposures and potential confounders | 7-8 | | The characteristics and clinical date of participants in the presence or absence of carotid plaque were presented in Table 1. | |
|  |  |  |  | (b) Indicate number of participants with missing data for each variable of interest |  | |  | |
|  |  |  |  | (c) *Cohort study*—Summarise follow-up time (eg, average and total amount) |  | |  | |
| Outcome data | | 15* | | *Cohort study*—Report numbers of outcome events or summary measures over time |  | |  | |
|  |  |  |  | *Case-control study—*Report numbers in each exposure category, or summary measures of exposure |  | |  | |
|  |  |  |  | *Cross-sectional study—*Report numbers of outcome events or summary measures | 9-10 | | As shown in Figure 1, the prevalence of carotid plaque were 34.9%, 43.4%, 45.2%, 54.0%, 61.8%, 67.2%, and 71.7% in patients with CHA_2_DS_2_-VASc score of 0, 1, 2, 3, 4, 5 and ≥ 6, respectively. The prevalence of carotid plaque increased significantly with the increase of CHA_2_DS_2_-VASc score in all participants, male participants and female participants (*P* for trend < 0.001). | |
| Main results | | 16 | | (*a*) Give unadjusted estimates and, if applicable, confounder-adjusted estimates and their precision (eg, 95% confidence interval). Make clear which confounders were adjusted for and why they were included | 10-11 | | Table 2 showed that per 1-point increase in CHA_2_DS_2_-VASc score was significantly associated with the prevalence of carotid plaque in all patients (OR: 1.304; 95% CI: 1.238-1.374), in male patients (OR: 1.537; 95% CI: 1.393-1.695) and in female patients (OR: 1.476; 95% CI: 1.372-1.588). | |
|  |  |  |  | (*b*) Report category boundaries when continuous variables were categorized |  | |  | |
|  |  |  |  | (*c*) If relevant, consider translating estimates of relative risk into absolute risk for a meaningful time period |  | |  | |
| Other analyses | 17 | | Report other analyses done—eg analyses of subgroups and interactions, and sensitivity analyses | |  | Not reported. | | |
| Discussion | | | | | | | | |
| Key results | 18 | | Summarise key results with reference to study objectives | | 12 | Our key findings were as follows: (1) CHA_2_DS_2_-VASc score was independent associated with carotid plaque in NVAF patients; (2) The optimal cutoff point of CHA_2_DS_2_-VASc score for predicting carotid plaque were 2 and 3 in male and female NVAF patients, respectively. | | |
| Limitations | 19 | | Discuss limitations of the study, taking into account sources of potential bias or imprecision. Discuss both direction and magnitude of any potential bias | | 14-15 | This study had several limitations. First, it was a single center, retrospective and observational study and had inherent limitations of a retrospective design. Second, the study population was searched from electronic medical record system, and had selective bias. Third, some risk factors which had been verified to be related to carotid plaque were not included as the database, which might lead to some bias in multivariate logistic regression analysis. Further prospective investigations are needed to confirm this correlation. | | |
| Interpretation | 20 | | Give a cautious overall interpretation of results considering objectives, limitations, multiplicity of analyses, results from similar studies, and other relevant evidence | | 14 | Our result suggested that CHA_2_DS_2_-VASc score was not only a marker of CE risk, but also a marker of non-CE risk in NVAF patients. NVAF patients with higher CHA_2_DS_2_-VASc score had a greater possibility of carotid plaque and atherosclerotic non-CE stroke risk. | | |
| Generalisability | 21 | | Discuss the generalisability (external validity) of the study results | | 14-15 | Therefore, further studies should be completed for more individualized prevention strategy in NVAF patients with carotid disease. | | |
| Other information | | |  | | | | | |
| Funding | 22 | | Give the source of funding and the role of the funders for the present study and, if applicable, for the original study on which the present article is based | | 1 | This study was supported by the National Natural Science Foundation of China (Grant number: 81570297, 81660053 and 81660071). | | |

*Give information separately for cases and controls in case-control studies and, if applicable, for exposed and unexposed groups in cohort and cross-sectional studies.

**Note:** An Explanation and Elaboration article discusses each checklist item and gives methodological background and published examples of transparent reporting. The STROBE checklist is best used in conjunction with this article (freely available on the Web sites of PLoS Medicine at http://www.plosmedicine.org/, Annals of Internal Medicine at http://www.annals.org/, and Epidemiology at http://www.epidem.com/). Information on the STROBE Initiative is available at www.strobe-statement.org.
